# Congenital Absence of Patella: A Case Report

**DOI:** 10.7759/cureus.48688

**Published:** 2023-11-12

**Authors:** Vineeta Pande, Mridu Bahal, Renuka Jadhav, Shailaja Mane, Jasleen Dua

**Affiliations:** 1 Pediatrics, Dr. D.Y. Patil Medical College, Hospital & Research Centre, Pune, IND

**Keywords:** knee joints, nail patella syndrome, gait disorder, congenital abnormalities, patella

## Abstract

Congenital absence of patella is a rare orthopedic condition characterized by an underdeveloped or complete lack of patella. This condition is very rare in isolation and is usually accompanied by other genetic syndromes. The prevalence is difficult to estimate as very few cases of this condition have been reported worldwide. Here, we report a case of congenital bilateral absence of patella in an 18-month-old female child who came with a chief complaint of inability to stand and walk without support, with hyperextension at the knee joint with no other associated abnormalities. The patient was started with active and resisted physiotherapy sessions that alleviated the condition of our patient.

## Introduction

The patella is the largest sesamoid bone found on the anterior aspect of the distal femur and contributes to forming the knee joint. It plays a critical role in improving the effectiveness of quadriceps extension capacity which is required to maintain normal gait and mobility [1]. Although the exact cause is mostly not clear, genetic predisposition has been attributed as a major contributing factor in many cases [2]. The prevalence rate is not readily available because the patella being completely cartilaginous at birth starts the ossification process between the third and sixth years of life.

It is mostly seen bilaterally and is associated with a clinically diverse and genetically heterogeneous group of disorders. Most commonly associated syndromes include Nail-patella syndrome, Turner-Kieser syndrome, ischiopatellar dysplasia (IPD), Meier-Gorlin syndrome, the RAPADILINO syndrome, the Genito patellar syndrome, and Trisomy 8q syndrome [3]. It is also occasionally associated with various orthopedic malformations, but the most common one is congenital dislocation of the knee or hip and anomalies of femur and fibula, genu recurvatum, genu valgum, cubitus valgus, and pelvic abnormality [4].

## Case presentation

**Figure 1:**
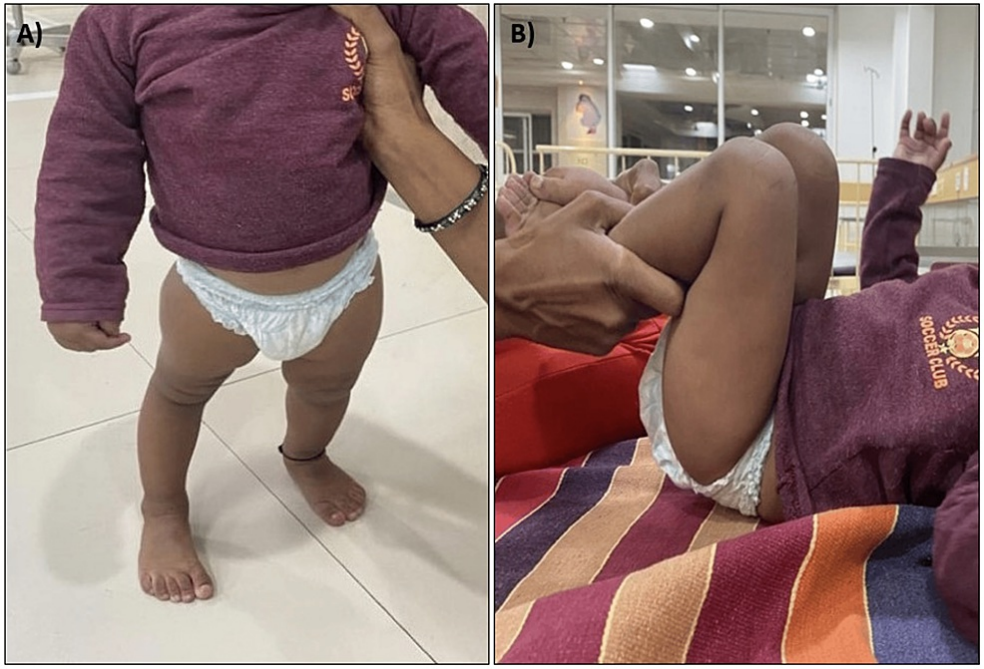
Clinical image of the patient showing A) hyperextension at the knee joint and B) complete flexion at the knee joint

**Figure 2:**
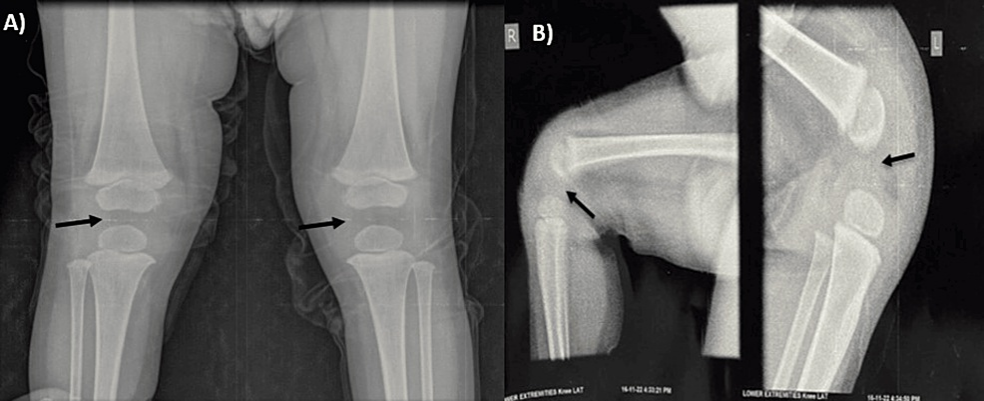
X-ray image confirming the absence of the patella Antero-posterior (A) and lateral (B) radiograph of bilateral knee. Arrows indicate the absence of the patella in the anterior aspect of the knee joint.

A female child, 18-month-old, came to the OPD with chief complaints of inability to stand and walk without support associated with inability to straighten the knee. The child was born from a non-consanguineous marriage at full term by normal delivery with knees deformed since birth and no history of affected siblings. The child was able to stand unaided for one to two minutes before falling, after which the child was only able to walk with support. All other developmental milestones were age-appropriate. On examination, double knee crease was present, on palpation patella was found to be absent, hyperextension (Figure 1A) and hypermobility were noted at the knee joint, and the patient was also able to do complete flexion (Figure 1B). The power and tone of quadriceps, gluteus maximus, and hamstrings were appropriate for age. Nails were also normal and no other deformity was reported in the patient. Systemic examination was within normal limits. Blood parameters were also normal in range. Ultrasonography of the abdomen and MRI brain were normal. X-rays confirmed the absence of the patella in both knees (Figure 2).

The child was started on active and resisted physiotherapy and was called for follow-up to assess the improvement in muscle strength, joint stability, and overall mobility. On follow-up, the patient was able to perform passive leg extension and was also able to stand for a longer duration without any support. 

## Discussion

Bilateral congenital absence of patella is a rare disorder that is associated with gait and mobility-related abnormalities. In some cases, as reported by Duygun et al. (2013), isolated unilateral absence of patella accompanied by contralateral small patella can also be found [3]. In the absence of any other osseous defect, severe lateral dislocation of the extensor mechanism occurs in conjunction with the congenital absence of patella [5]. In such cases or when conservative treatments are ineffective, successful placement of the mechanism in the groove between the femoral condyles, medialization of the tibial tuberosity, and transfer of one or more medial hamstring tendons to the extensor mechanism are the preferred treatments resulting in satisfactory functioning [6]. The tibial tuberosity and the femoral condyles are, in the majority of cases, larger than normal. The well-developed extensor mechanism glides between femoral condyles in the patellar groove. Reports have suggested that strengthening muscles around the knee joint can be pivotal in the management plan [7]. Jerome et al. (2008) also concur that regular physiotherapy and stretching exercises can alleviate the deformity and improve the gait of patients [4]. Considering the previous reports and the satisfactory outcomes, we also started active and resisted physiotherapy. The child showed improvement and was able to stand for a longer duration without any support. 

## Conclusions

Bilateral congenital absence of patella causes gait and mobility-related abnormalities and has a severe impact on the patient’s quality of life. Treatment is directed toward supportive care to prevent major disability in patients. With early diagnosis and multimodal treatment, including physiotherapy and reconstructive surgical intervention where necessary, patients with this condition can lead a fulfilling life with improved joint function and reduced abnormalities.
